# Antimicrobial Susceptibility of *Pseudomonas aeruginosa* from Elderly Patients in Intensive Care Units of United States Medical Centers (2021–2025)

**DOI:** 10.3390/antibiotics15040361

**Published:** 2026-04-01

**Authors:** Helio S. Sader, Rodrigo E. Mendes, Timothy B. Doyle, Marisa L. Winkler, Mariana Castanheira

**Affiliations:** Element Iowa City (JMI Laboratories), North Liberty, IA 52317, USA

**Keywords:** ceftazidime-avibactam, ceftolozane-tazobactam, imipenem-relebactam, multidrug-resistant, *Pseudomonas aeruginosa*

## Abstract

**Objectives**: The primary objective was to evaluate the antimicrobial susceptibility of *Pseudomonas aeruginosa* causing infection in elderly (≥65 years old) patients hospitalized in intensive care units (ICUs) of United States medical centers. Susceptibility results from isolates of elderly patients in ICUs were compared to isolates from elderly patients not in ICUs (elderly non-ICU) and adult ICU patients (18 to 64 years old; adult ICU). **Methods**: *P. aeruginosa* isolates were consecutively collected from 74 US medical centers in 2021–2025 and susceptibility tested by reference broth microdilution in the monitoring laboratory (Element Iowa City [JMI Laboratories]). The organism collection included 999 isolates from elderly ICU, 2027 isolates from elderly non-ICU, and 1022 isolates from adult ICU patients. **Results**: The most active agents against *P. aeruginosa* from all three patient groups were ceftazidime-avibactam (95.8% to 97.3% susceptible), ceftolozane-tazobactam (96.0% to 98.3% susceptible), imipenem-relebactam (97.6% to 98.7% susceptible), and tobramycin (91.4% to 94.7% susceptible). Susceptibility to piperacillin-tazobactam, ceftazidime, cefepime, meropenem, and imipenem were markedly lower among isolates from elderly and adult ICU patients compared to elderly non-ICU patients. Susceptibility to levofloxacin and tobramycin were lower among isolates from adult ICU patients compared to elderly ICU and non-ICU patients. Moreover, the frequency of multidrug-resistant (MDR) isolates was markedly higher among elderly (18.4%) and adult (22.4%) ICU patients compared to elderly non-ICU (11.0%) patients. An annual analysis of susceptibility to selected β-lactams showed a slight variation in susceptibility rates without a clear trend. **Conclusions**: Ceftazidime-avibactam, ceftolozane-tazobactam, and imipenem-relebactam were highly active and exhibited similar coverage against a large contemporary collection of *P. aeruginosa* isolates from ICU elderly, non-ICU elderly, and ICU adult patients. Cross-resistance among these β-lactamase inhibitor combinations (BLICs) varied markedly, indicating that all three should be tested in the clinical laboratory and available for clinical use.

## 1. Introduction

*P. aeruginosa* infections represent a particular challenge when treating elderly and critically ill patients [1,2]. A key factor that contributes to poor clinical outcomes are delays before starting effective antimicrobial therapy, which is more common among patients infected with organisms resistant to antimicrobial agents than among patients infected with susceptible organisms [3,4]. Moreover, many factors are responsible for increasing antimicrobial resistance in elderly patients hospitalized in ICUs, including comorbidities, invasive procedures, use of indwelling devices, prolonged hospital stays, and high use of antimicrobial agents [3,5].

The following antimicrobial classes with clinically meaningful antipseudomonal activity are approved by the US Food and Drug Administration (FDA) for treating systemic *P. aeruginosa* infections: cephalosporins (ceftazidime, cefepime, and cefiderocol), monobactam (aztreonam), carbapenems (imipenem and meropenem), β-lactamase inhibitor combinations (BLICs; piperacillin-tazobactam, ceftolozane-tazobactam, ceftazidime-avibactam, and imipenem-relebactam), fluoroquinolones (ciprofloxacin and levofloxacin), aminoglycosides (gentamicin, tobramycin, and amikacin), and lipopeptides (colistin and polymyxin B). The approved indications for each of these agents vary markedly [6]. Despite their potential *in vitro* activity, colistin and polymyxin B are considered inappropriate therapy for systemic *P. aeruginosa* infection. In addition to nephrotoxicity, the penetration of these lipopeptides into pulmonary tissue limits their effectiveness in pneumonia [7]. Meropenem-vaborbactam is approved for the treatment of *P. aeruginosa* infections by the European Medicine Agency (EMA), but not by the US FDA, and it is important to note that vaborbactam does not improve meropenem’s activity against *P. aeruginosa* [6,8].

The objective of this investigation was to evaluate the antimicrobial susceptibility of *P. aeruginosa* causing infection in elderly patients (≥65 years old) hospitalized in the ICUs (elderly ICU) of US medical centers. Susceptibility results of isolates from elderly ICU patients were compared to those from elderly patients hospitalized in other wards (elderly non-ICU) and from adults (18 to 64 years old; adult ICU) hospitalized in the same ICUs as the elderly patients during the same period.

## 2. Results

**Figure 1:**
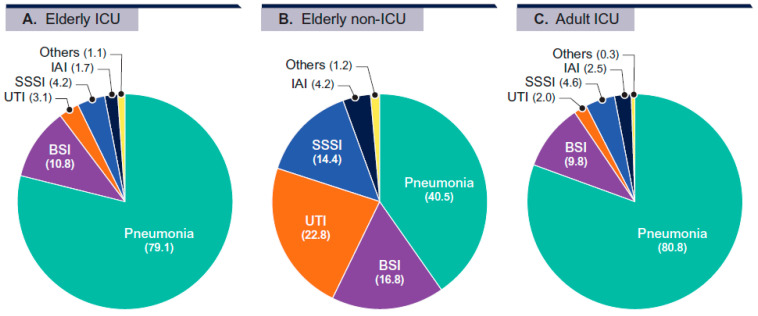
Distribution of isolates by infection site.

*P. aeruginosa* isolates from elderly ICU patients were largely from patients with pneumonia (79.1%) and bloodstream infection (BSI; 10.8%), whereas isolates from elderly non-ICU patients were predominantly from pneumonia (40.5%), urinary tract infection (UTI; 22.8%), and BSI (16.8%; Figure 1). *P. aeruginosa* isolates from adults in the ICU were mostly from patients with pneumonia (80.8%) and BSI (9.8%; Figure 1).

**Figure 2:**
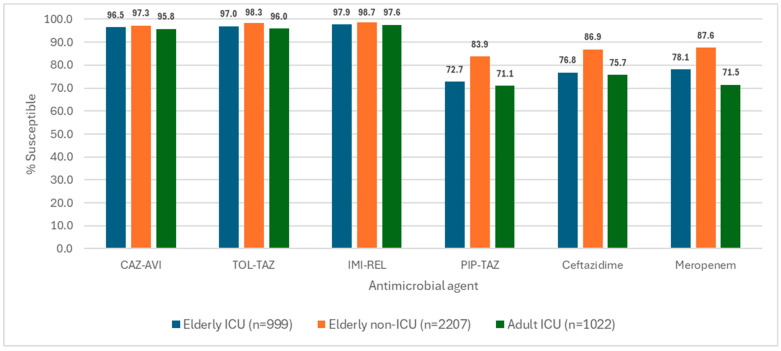
Antimicrobial susceptibility of selected agents. Abbreviations: CAZ-AVI, ceftazidime-avibactam; TOL-TAZ, ceftolozane-tazobactam; IMI-REL, imipenem-relebactam; and PIP-TAZ, piperacillin-tazobactam.

The most active agents against *P. aeruginosa* from all three patient groups (elderly ICU, elderly non-ICU, and adult ICU patients) were ceftazidime-avibactam (95.8% to 97.3% susceptible), ceftolozane-tazobactam (96.0% to 98.3% susceptible), and imipenem-relebactam (97.6% to 98.7% susceptible; Table 1 and Figure 2). These three BLICs were very active against isolates from these three patient groups, with susceptibility rates slightly higher among isolates from elderly non-ICU patients (97.3% to 98.7%) compared to elderly (96.5% to 97.9%) and adult ICU patients (95.8% to 97.6%; Table 1 and Figure 2).

**Figure 3:**
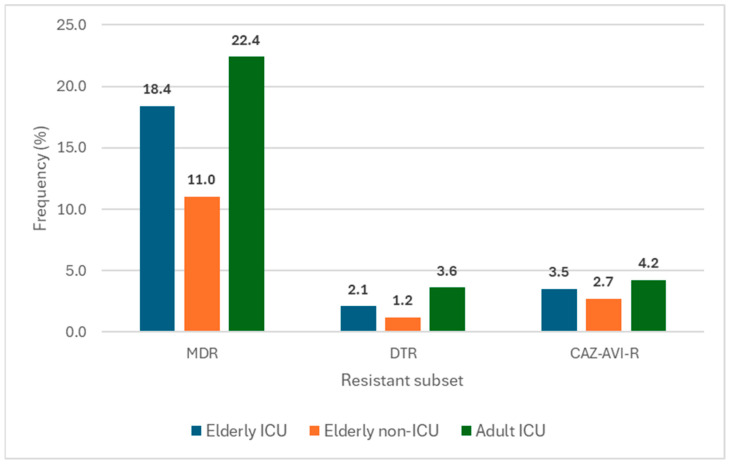
Frequencies of multidrug-resistant (MDR), difficult-to-treat resistant (DTR), and ceftazidime-avibactam-resistant (CAZ-AVI-R) organisms.

Susceptibility to piperacillin-tazobactam, ceftazidime, cefepime, meropenem, and imipenem were significantly lower among isolates from elderly ICU patients compared to elderly non-ICU patients (*p* < 0.001 for all agents listed above) and slightly lower among isolates from adult compared to elderly ICU patients (Table 1). Susceptibility to levofloxacin and tobramycin were similar between elderly ICU and non-ICU patients, while isolates from adult ICU patients showed lower susceptibility to these two agents when compared to elderly patients from both ICU and non-ICU clinical settings (Table 1). Most importantly, the frequency of MDR isolates was significantly higher among elderly ICU (18.4%; *p* < 0.001 [OR: 1.478–2.258]) and adult ICU (22.4%; *p* < 0.001 [OR: 1.908–2.860]) patients compared to elderly non-ICU (11.0%) patients. Similarly, the frequency of difficult-to-treat resistant (DTR) isolates was markedly higher among elderly (2.1%; *p* = 0.066 [OR: 0.958–3.087]) and adult (3.6%; *p* < 0.001 [OR: 1.801–5.025]) ICU patients when compared to elderly non-ICU patients (1.2%; Figure 3).

When isolates from patients with pneumonia were analyzed separately, the BLICs ceftazidime-avibactam, ceftolozane-tazobactam, and imipenem-relebactam retained potent activity (>95% susceptible) and exhibited similar susceptibility rates against *P. aeruginosa* isolates from all three patient groups (Table 1). Susceptibility to piperacillin-tazobactam, ceftazidime, cefepime, meropenem, imipenem, levofloxacin, and tobramycin were lower among isolates from elderly ICU compared to non-ICU elderly patients and were lower among isolates from adult ICU compared to elderly ICU patients (Table 1).

Only the new BLICs ceftazidime-avibactam, ceftolozane-tazobactam, imipenem-relebactam, and tobramycin exhibited good activity against MDR and difficult-to-treat (DTR) *P. aeruginosa*. The most active agent against MDR *P. aeruginosa* from elderly ICU patients was imipenem-relebactam (MIC_50/90_, 1/4 mg/L; 88.8% susceptible), followed by ceftolozane-tazobactam (MIC_50/90_, 2/8 mg/L; 83.7% susceptible), ceftazidime-avibactam (MIC_50/90_, 4/16 mg/L; 81.0% susceptible), and tobramycin (MIC_50/90_, 0.5/4 mg/L; 76.1% susceptible; Table 1). The most active agents against DTR *P. aeruginosa* from elderly ICU patients were ceftolozane-tazobactam (MIC_50/90_, 2/8 mg/L; 85.7% susceptible), tobramycin (MIC_50/90_, 1/2 mg/L; 81.0% susceptible), ceftazidime-avibactam (MIC_50/90_, 8/16 mg/L; 71.4% susceptible), and imipenem-relebactam (MIC_50/90_, 2/4 mg/L; 71.4% susceptible; Table 1). Meropenem-vaborbactam inhibited 55.5% to 64.6% of MDR isolates and 24.3% to 42.9% of DTR isolates at the European Committee on Antimicrobial Susceptibility Testing (EUCAST) susceptible breakpoint of ≤8/8 mg/L (Table 1). Yearly analysis of susceptibility to selected β-lactams showed slight variation in susceptibility rates without a clear trend of increase or decrease over the years of the investigation (Table 2). Frequencies of MDR and DTR phenotypes showed a larger yearly variation, but again without a clear trend of increase or decrease over the years of the investigation (Table 3). Notably, we did not detect any pan-drug resistant isolate in this study.

Notably, ceftazidime-avibactam maintained *in vitro* activity against 33.8% and 59.1% of isolates not susceptible to ceftolozane-tazobactam and imipenem-relebactam, respectively. Ceftolozane-tazobactam retained activity against 52.2% and 60.5% of isolates not susceptible to ceftazidime-avibactam and imipenem-relebactam, respectively. Moreover, imipenem-relebactam remained active against 79.3% and 72.1% of isolates not susceptible to ceftazidime-avibactam and ceftolozane-tazobactam, respectively (Table 4).

## 3. Discussion

*P. aeruginosa* is intrinsically resistant to many antibiotics and has a great ability to acquire or develop additional mechanisms to overcome multiple classes of antimicrobial agents. Therefore, *P. aeruginosa* poses a serious therapeutic challenge, and prompt initiation of effective antimicrobial therapy is essential to optimize clinical outcomes [1,2,3,6,8,9].

We evaluated the antimicrobial susceptibility of >4000 *P. aeruginosa* isolates from three groups: elderly ICU (*n* = 999), elderly non-ICU (*n* = 2027), and adult ICU (*n* = 1022) patients. Our results showed that the BLICs ceftazidime-avibactam, ceftolozane-tazobactam, and imipenem-relebactam continue to be very active against *P. aeruginosa* isolates causing infections in these patients.

Ceftazidime-avibactam and ceftolozane-tazobactam were approved for clinical use by the US FDA in late 2014 (ceftolozane-tazobactam) and early 2015 (ceftazidime-avibactam), and the approval of these agents represented a significant improvement in the treatment of *P. aeruginosa* infections [10,11]. Although these agents have been clinically used for more than 10 years, the results of this investigation demonstrate that they remain highly active against *P. aeruginosa*, including those causing infection in elderly and ICU patients.

Imipenem-relebactam was initially approved by the US FDA in July 2019 (for complicated UTI and complicated intra-abdominal infections in adults), with an expanded approval in June 2020 (for hospital-acquired and ventilator-associated bacterial pneumonia in adults) [12], and showed *in vitro* activity similar to ceftazidime-avibactam and ceftolozane-tazobactam. It is important to observe that resistance to these three BLICs did not increase during the 5-year study period (2021–2025). Also, isolates resistant to one of these BLICs may remain susceptible to one or both other BLICs. For instance, imipenem-relebactam remained active against 72.1% to 79.3% of isolates not susceptible to ceftazidime-avibactam or ceftolozane-tazobactam, whereas ceftazidime-avibactam and ceftolozane-tazobactam were active against approximately 60% of isolates not susceptible to imipenem-relebactam. In summary, cross-resistance among these BLICs varied markedly, indicating that all three should be tested and available for clinical use.

Our results also indicate that resistance to other β-lactams commonly used to treat *P. aeruginosa* infections, such as piperacillin-tazobactam, carbapenems (meropenem and imipenem), and cephalosporins (ceftazidime and cefepime), as well as the frequencies of MDR and DTR isolates, were higher among isolates from ICU patients (both elderly and adult patients) when compared to elderly non-ICU patients. The fact that resistance to these β-lactams was higher among both elderly and adult patients from the ICU compared to elderly non-ICU patients may indicate that increased resistance to β-lactams is more related to ICU conditions than to factors associated with age. In contrast, resistance to levofloxacin and tobramycin were higher among adult ICU compared to elderly ICU patients and similar between the two groups (ICU and non-ICU) of elderly patients. One important limitation of this study was the lack of evaluation of possible factors related to the differences in antimicrobial resistance rates among isolates from the three patient groups. Unfortunately, the INFORM program does not have access to patient data that could be linked to antimicrobial resistance, such as previous antimicrobial use, comorbidities, or invasive procedures [13].

Because the frequency of pneumonia was markedly higher among ICU patients (both elderly and adult patients) compared to elderly non-ICU patients, and isolates from pneumonia have been related to higher antimicrobial resistance compared to other infections [14], we analyzed isolates from pneumonia separately. Notably, similar differences in antimicrobial resistance rates among patient groups were observed with isolates from pneumonia compared to isolates from all infection types combined.

We could not find other studies that evaluated the antimicrobial susceptibility of *P. aeruginosa* in elderly ICU patients to compare with our results. Quiang et al. evaluated 2208 *P. aeruginosa* isolates from 75 hospitals across Hebei Province, China, between 2016 and 2021. The median age of patients with *P. aeruginosa* was 63 years. The results of their study indicate that *P. aeruginosa* bloodstream infections primarily affected hematology and ICU patients, mainly those who were middle-aged, elderly, and male. Notably, the investigators observed an overall downward trend in antimicrobial resistance from 2016 to 2021, with variations in resistance patterns across hospital units and age groups [15].

Mesquita et al. assessed antimicrobial resistance of *P. aeruginosa* from patients with pneumonia during the COVID-19 pandemic and pre-pandemic periods in Northeast Brazil and observed an increase in both the frequency of *P. aeruginosa* infections and antimicrobial resistance in elderly patients [16]. Golli et al. observed high resistance rates overall and increasing resistance to carbapenems among *Acinetobacter* spp. and *Klebsiella* spp. from elderly patients hospitalized in the ICU of a Romanian hospital between 2022 and 2024 [17].

Moreover, our group previously analyzed and reported the antimicrobial susceptibility of *P. aeruginosa* isolates collected from ICU and non-ICU patients (all ages) in 72 US medical centers from 2020–2022 [18]. Ceftazidime-avibactam was active against 96.3% of ICU isolates and 97.6% of non-ICU isolates, and ceftolozane-tazobactam was active against 97.2% of ICU isolates and 98.4% of non-ICU isolates, which are similar to the results obtained with isolates from elderly patients in the present study. Susceptibility rates for ceftolozane-tazobactam (97.2%/98.4% for ICU/non-ICU), imipenem-relebactam (97.1%/98.0% for ICU/non-ICU), meropenem-vaborbactam (90.0%/94.3% for ICU/non-ICU), meropenem (76.9%/85.8% for ICU/non-ICU) and imipenem (77.4%/84.3% for ICU/non-ICU) were also similar in both studies. In contrast, susceptibility rates for piperacillin-tazobactam (77.8%/84.6% for ICU/non-ICU), ceftazidime (81.4%/87.8% for ICU/non-ICU), and cefepime (84.7%/89.0% for ICU/non-ICU) were higher, and susceptibility rates for levofloxacin (73.3%/73.2% for ICU/non-ICU) and tobramycin (92.1%/92.1% for ICU/non-ICU) were slightly lower in the previous study [18] when compared to the present investigation. In summary, many factors affect the antimicrobial susceptibility of *P. aeruginosa* and resistance rates may vary widely among geographic regions, patient ages, infection types, and hospital units [19].

## 4. Methods

### 4.1. Organism Collection

Bacterial isolates were obtained through the International Network for Optimal Resistance Monitoring (INFORM) program [12]. Seventy-four US medical centers contributed isolates between January 2021 and December 2025. According to a common study protocol, medical centers collected a defined number of consecutive isolates (one per infection episode) from designated infection types, independent of bacterial species or hospital unit. We assessed the antimicrobial susceptibility of *P. aeruginosa* isolates from three groups of patients: (i) elderly patients (≥65 years old) hospitalized in ICUs (*n* = 999), (ii) elderly patients hospitalized in other (non-ICU) units (*n* = 2027), and (iii) adults (18 to 64 years old) hospitalized in the same ICUs as the elderly patients during the same period (*n* = 1022). Isolates were considered clinically significant by algorithms established by the participant medical centers.

### 4.2. Susceptibility Testing

Susceptibility testing was performed by the broth microdilution method using cation-adjusted Muller–Hinton media (Becton and Dickson Company [BD]; Franklin Lakes, NJ, USA), as described by the Clinical Laboratory Standard Institute (CLSI) [20]. CLSI and/or US FDA breakpoint criteria were used to interpret MIC values unless noted [20,21]. Neither the CLSI nor the US FDA have published breakpoint criteria for meropenem-vaborbactam against *P. aeruginosa* because this compound is not approved for treatment of *P. aeruginosa* infections in the US; thus, EUCAST criteria [22] were applied for this organism–drug combination for comparison purposes. MDR criteria were defined as nonsusceptibility to ≥1 agent in ≥3 antimicrobial classes according to criteria defined in 2012 by the joint European and US Centers for Disease Control [23]. The antimicrobial classes, representative agents and corresponding interpretive criteria for nonsusceptibility were: (1) anti-*Pseudomonas* cephalosporins and monobactams (ceftazidime [≥16 mg/L], cefepime [≥16 mg/L], and aztreonam [≥16 mg/L]); (2) carbapenems (meropenem [≥4 mg/L] and imipenem [≥4 mg/L]); (3) old BLICs (piperacillin/tazobactam [≥32/4 mg/L]); (4) new BLICs (ceftazidime-avibactam [≥16/4 mg/L], ceftolozane-tazobactam [≥8/4 mg/L], and imipenem-relebactam [≥4/4 mg/L]); (5) aminoglycosides (tobramycin [≥2 mg/L]); (6) fluoroquinolones (levofloxacin [≥2 mg/L] and ciprofloxacin [≥1 mg/L]); and (7) lipopeptides (colistin [≥4 mg/L]) [23]. DTR resistance was defined as nonsusceptibility to piperacillin-tazobactam, ceftazidime, cefepime, meropenem, imipenem, levofloxacin, and ciprofloxacin [24].

### 4.3. Statistical Analysis

The chi-square test was applied to find significant differences between two groups. Statistical analyses were performed with the Epi Info TM 7 statistical package version 7.2 (United States Centers for Disease Control and Prevention, Atlanta, GA, USA). A *p* value of <0.05 was considered statistically significant.

## 5. Conclusions

The main findings of this investigation were as follows: (1) The BLICs ceftazidime-avibactam, ceftolozane-tazobactam, and imipenem-relebactam remained very active against *P. aeruginosa* causing infection in elderly ICU, elderly non-ICU, and adult ICU patients; (2) Resistance to other β-lactams commonly used to treat *P. aeruginosa* infections was higher among elderly ICU and adult ICU patients than it was in elderly non-ICU patients; and (3) Resistance to levofloxacin and tobramycin was higher among adult ICU patients than it was in elderly ICU and elderly non-ICU patients. The results of this investigation expand the results of other reports and could help guide empirical therapy for systemic *P. aeruginosa* infections. Large surveillance programs are essential to monitor the *in vitro* activity and guide the clinical use of antimicrobial agents.

## Figures and Tables

**Table 1:** Antimicrobial susceptibility rates of *P. aeruginosa* isolates from elderly patients hospitalized in ICU and non-ICU units and from adults hospitalized in ICU units (US hospitals; 2021–2025).

*P. aeruginosa* Subset/	% Susceptible (No. of Isolates Tested) ^a^		
Antimicrobial Agent	Elderly		Adults
	ICU	Non-ICU	ICU
All *P. aeruginosa*	(999)	(2027)	(1022)
Ceftazidime-avibactam	96.5	97.3	95.8
Ceftolozane-tazobactam	97.0	98.3	96.0
Imipenem-relebactam	97.9	98.7	97.6
Meropenem-vaborbactam ^b^	91.8 ^b^	95.4 ^b^	87.9 ^b^
Piperacillin-tazobactam	72.7	83.9	71.1
Ceftazidime	76.8	86.9	75.7
Cefepime	81.2	88.8	79.1
Meropenem	78.1	87.6	71.5
Imipenem	78.3	85.5	72.4
Levofloxacin	76.4	77.3	68.3
Tobramycin	93.9	94.7	91.4
Isolates from pneumonia	(790)	(821)	(826)
Ceftazidime-avibactam	96.6	95.1	96.2
Ceftolozane-tazobactam	96.3	96.7	96.2
Imipenem-relebactam	97.9	98.5	97.4
Meropenem-vaborbactam ^b^	91.5 ^b^	93.4 ^b^	87.2 ^b^
Piperacillin-tazobactam	71.4	78.9	70.1
Ceftazidime	75.9	81.5	74.6
Cefepime	79.9	84.1	77.9
Meropenem	76.2	83.6	69.6
Imipenem	77.3	81.5	70.1
Levofloxacin	76.1	76.0	66.8
Tobramycin	93.4	92.3	90.3
MDR *P. aeruginosa*	(184)	(223)	(229)
Ceftazidime-avibactam	81.0	77.1	81.7
Ceftolozane-tazobactam	83.7	84.7	82.1
Imipenem-relebactam	88.8	89.4	89.1
Meropenem-vaborbactam ^b^	62.0 ^b^	64.6 ^b^	55.5 ^b^
Piperacillin-tazobactam	12.0	13.5	13.5
Ceftazidime	20.7	27.4	30.1
Cefepime	27.7	29.6	28.8
Meropenem	17.9	27.4	16.2
Imipenem	28.3	34.5	27.1
Levofloxacin	31.5	23.3	25.8
Tobramycin	76.1	74.4	73.8
DTR *P. aeruginosa*	(21)	(25)	(37)
Ceftazidime-avibactam	71.4	60.0	62.2
Ceftolozane-tazobactam	85.7	80.0	70.3
Imipenem-relebactam	71.4	72.7	68.8
Meropenem-vaborbactam ^b^	42.9	32.0	24.3
Tobramycin	81.0	60.0	67.6

Abbreviations: MDR, multi-drug resistant; DTR, difficult-to-treat resistant. ^a^ Susceptibility per CLSI and/or US FDA criteria. ^b^ This compound is not approved for treatment of *P. aeruginosa* infections in the US. EUCAST criteria were applied, as neither the CLSI nor US FDA publish breakpoint criteria.

**Table 2:** Yearly susceptibility rates for selected β-lactam compounds.

Patient Group/	% Susceptible per CLSI (No. of Isolates)				
Antimicrobial Agent	2021	2022	2023	2024	2025
ICU Elderly Patients	(196)	(178)	(219)	(205)	(201)
Ceftazidime-avibactam	97.4	97.2	96.3	95.1	96.5
Ceftolozane-tazobactam	98.5	97.8	95.0	95.6	98.5
Imipenem-relebactam	95.3	98.3	98.6	99.5	97.5
Piperacillin-tazobactam	73.0	82.0	69.9	72.2	67.7
Meropenem	77.0	79.2	74.4	80.5	79.6
Non-ICU Elderly Patients	(441)	(439)	(399)	(344)	(404)
Ceftazidime-avibactam	97.3	97.7	97.5	98.0	96.0
Ceftolozane-tazobactam	97.5	98.6	98.7	99.1	97.5
Imipenem-relebactam	98.2	98.6	99.2	99.4	98.0
Piperacillin-tazobactam	85.7	84.5	83.7	81.1	83.7
Meropenem	89.1	86.6	87.5	86.9	87.6
ICU Adult Patients	(222)	(188)	(236)	(208)	(168)
Ceftazidime-avibactam	94.1	94.7	96.6	98.1	95.2
Ceftolozane-tazobactam	95.9	94.7	96.6	96.6	95.8
Imipenem-relebactam	94.3	98.4	98.3	98.6	98.2
Piperacillin-tazobactam	73.4	75.5	69.5	66.8	70.8
Meropenem	71.2	71.8	78.8	70.7	62.5

**Table 3:** Yearly frequency of resistance subsets.

Patient Group/	Frequency (No. of Isolates Tested)					
Resistant Subset	2021	2022	2023	2024	2025	Overall
ICU Elderly Patients	(196)	(178)	(219)	(205)	(201)	(999)
MDR	18.4	13.5	21.0	17.6	20.9	18.4
DTR	2.0	1.7	1.4	1.5	4.0	2.1
Non-ICU Elderly Patients	(441)	(439)	(399)	(344)	(404)	(2027)
MDR	9.3	12.1	11.0	10.8	11.9	11.0
DTR	1.6	1.8	1.3	0.9	0.5	1.2
ICU Adult Patients	(222)	(188)	(236)	(208)	(168)	(1022)
MDR	23.0	17.0	20.3	26.0	26.2	22.4
DTR	6.8	3.7	2.1	2.9	2.4	3.6

**Table 4:** Cross resistance rates among β-lactamases inhibitor combinations when testing *P. aeruginosa* isolates from elderly patients.

	% Susceptible by Resistance Phenotype (No. of Isolates)		
Antimicrobial Agent	Ceftazidime-Avibactam Resistant (90) ^a^	Ceftolozane-Tazobactam Nonsusceptible (65) ^a^	Imipenem-Relebactam Nonsusceptible (44) ^a^
Ceftazidime-avibactam	0.0	33.8	59.1
Ceftolozane-tazobactam	52.2	0.0	60.5
Imipenem-relebactam	79.3	72.1	0.0

^a^ Includes all isolates (ICU and non-ICU) from elderly patients.

## Data Availability

The original contributions presented in this study are included in the article. Further inquiries can be directed to the corresponding author.
